# Immunomodulatory Empty/Hollow Nanoparticles as Potential Therapeutic Strategies for Septic Shock

**DOI:** 10.3390/biomedicines14071460

**Published:** 2026-06-27

**Authors:** Gracy Xavier Rosario, Gelilla Daniel, Philemon Shallie, Danielle Kinsey, Nathan Carpenter, Othman Sheikh Hussein, Cuthbert Ormond Simpkins

**Affiliations:** 1 Department of Surgery, School of Medicine, University of Missouri, Kansas City, MO 64108, USA; pdsxfn@umkc.edu (P.S.); coscbc@umkc.edu (C.O.S.); 2 Vivacelle Bio Inc., Kansas City, MO 64108, USA; gelilladaniel@gmail.com (G.D.); doctordaniellekinsey@gmail.com (D.K.); njc6f7@umkc.edu (N.C.); osheikhhussein@gmail.com (O.S.H.)

**Keywords:** hollow nanoparticles, septic shock, immunomodulation, macrophages, VBI-S

## Abstract

Septic shock is a life-threatening manifestation of sepsis characterized by dysregulated immune responses, excessive inflammation, oxidative stress, and progressive multi-organ dysfunction. Despite advances in antimicrobial therapy and supportive care, mortality remains high, highlighting the need for therapeutic strategies that target immune dysregulation in addition to infection control. The review evaluates the potential of hollow nanoparticles as immunomodulatory therapies for septic shock, focusing on lipid-based, polymeric, protein-based, biomimetic, inorganic, carbon-based, and hybrid nanoparticle platforms. Current evidence suggests that these systems can modulate key pathological processes through reactive oxygen and nitrogen species (RONS) scavenging, regulation of inflammatory signaling, macrophage modulation, neutralization of bacterial toxins and antigens, and, in some cases, direct antimicrobial activity. Among the available platforms, lipid-based and biomimetic nanoparticles appear to possess the greatest translational potential owing to their favorable immunomodulatory properties and improved biocompatibility. Nonetheless, several challenges continue to limit clinical translation, including nanoparticle-associated systemic and organ toxicity, unintended immunogenicity, limited long-term safety data, and the lack of standardized comparative studies across nanoparticle classes. Despite these limitations, the progression of VBI-S, a phospholipid nanoparticle formulation, to Phase III clinical evaluation highlights the growing clinical feasibility of such nanoparticle-based approaches for septic shock. Future research should focus on optimizing nanoparticle design, improving safety profiles, and establishing standardized preclinical and clinical evaluation frameworks. Collectively, the available evidence suggests that hollow nanoparticles represent a promising antibiotic-independent strategy for restoring immune homeostasis and improving outcomes in septic shock.

## 1. Introduction

Sepsis is a life-threatening syndrome characterized by acute organ dysfunction resulting from a dysregulated host response to infection caused by bacteria, fungi, viruses, or parasites [1,2]. According to the World Health Organization, sepsis affects approximately 49 million people worldwide each year and is responsible for nearly 11 million deaths annually, highlighting its substantial global health burden [3,4]. In the United States, the Centers for Disease Control and Prevention (CDC) estimates that approximately 1.7 million adults develop sepsis annually, with the highest incidence and mortality occurring among individuals aged 65 years and older [1,5]. Although sepsis affects millions of individuals worldwide, only a subset of patients progresses to septic shock, the most severe form of the disease. Septic shock is characterized by profound circulatory and metabolic abnormalities, persistent hypotension requiring vasopressor support, and a markedly increased risk of mortality. Despite advances in antimicrobial therapy, fluid resuscitation, and vasopressor management, mortality rates remain high, often exceeding 30–40%. These limitations underscore the need for novel therapeutic approaches that target the underlying immune dysregulation driving disease progression. Consequently, hollow nanoparticles have emerged as promising immunomodulatory platforms with the potential to complement existing therapies and improve outcomes in patients with septic shock.

The pathophysiology of sepsis involves the simultaneous activation of pro- and anti-inflammatory signaling pathways in response to infection, resulting in widespread tissue injury and progressive immune dysfunction. When this dysregulated immune response is not adequately controlled, it can progress to severe sepsis, a condition characterized by excessive inflammatory signaling and subsequent immune dysfunction. Elevated levels of pro-inflammatory mediators, including tumor necrosis factor (TNF) and interleukin-1 (IL-1), trigger complex downstream signaling cascades that contribute to cytokine storm, myocardial depression, and multi-organ dysfunction syndrome (MODS). As the disease progresses, septic shock develops and is characterized by persistent hypotension despite adequate fluid resuscitation, worsening organ dysfunction, and a substantially increased risk of mortality [6].

Despite advances in critical care, there remains no definitive curative therapy for severe sepsis and septic shock. Current clinical management relies on early recognition, prompt administration of broad-spectrum antibiotics, aggressive fluid resuscitation, source control, and supportive interventions aimed at limiting organ injury and improving survival outcomes [7]. However, the increasing prevalence of antimicrobial resistance has significantly compromised the effectiveness of antibiotic-based therapies, particularly given the narrow therapeutic window for timely and appropriate antimicrobial selection. In recent years, multidrug-resistant ESKAPEE pathogens, including *Enterococcus faecium*, *Staphylococcus aureus*, *Klebsiella pneumoniae*, *Acinetobacter baumannii*, *Pseudomonas aeruginosa*, *Enterobacter* spp., and *Escherichia coli*, have emerged as major contributors to sepsis-associated morbidity and mortality [8]. The growing ability of these pathogens to acquire and deploy diverse resistance mechanisms has progressively reduced the efficacy of conventional antibiotic regimens, thereby limiting treatment success [9]. Consequently, there is an urgent need to develop innovative therapeutic and diagnostic strategies that can reduce reliance on antibiotics and more effectively target the host–pathogen interactions underlying sepsis.

Nanomedicine-based approaches, including hollow nanoparticle platforms, have recently attracted considerable attention as potential therapeutic strategies for sepsis and septic shock because of their antimicrobial and immunomodulatory properties. Accordingly, this review provides a comprehensive evaluation of hollow nanotechnologies with potential applications in the treatment of sepsis and septic shock. We discuss the mechanisms through which hollow nanoparticles modulate immune responses, attenuate excessive inflammation and oxidative stress-mediated tissue injury and neutralize pathogens and pathogen-derived antigens that contribute to disease progression and mortality. Furthermore, we summarize the available preclinical evidence and emerging clinical data supporting their therapeutic potential. The review also critically examines the safety considerations, current limitations, and translational challenges associated with these nanoplatforms while highlighting key areas for future investigation. Collectively, this review aims to provide a balanced perspective on the opportunities and challenges associated with hollow nanoparticle-based therapies and to guide the development of next-generation nanomedicines for sepsis and septic shock, conditions for which effective U.S. Food and Drug Administration (FDA)-approved therapeutic options remain limited.

## 2. Immune Pathophysiology of Sepsis

In sepsis and septic shock, microbial infections, predominantly caused by bacteria and, to a lesser extent, by viruses, parasites, and fungi, trigger the release of immunological mediators that, if not adequately controlled, result in excessive inflammation, vascular dysfunction, and progressive organ injury. Sepsis may also arise from non-infectious conditions, such as traumatic injury, which can induce systemic inflammatory response syndrome (SIRS) and subsequent organ damage [2,4,9]. In response to infection, the innate immune system initiates adaptive immune responses within the host [2]. Monocytes differentiate into macrophages, and neutrophils are recruited to sites of infection. Upon activation by bacterial products, macrophages acquire microbicidal properties and attempt to eliminate pathogens through phagocytosis [10]. However, bacteria can evade host defense mechanisms through biofilm formation, encapsulation, the generation of L-forms, and planktonic growth [2]. Thus, the development of sepsis is determined by the balance between microbial virulence mechanisms and host immune defenses.

The immune system recognizes invading pathogens through pattern recognition receptors (PRRs), which are expressed either intracellularly or on the cell surface of antigen-presenting cells such as dendritic cells, monocytes and macrophages. These receptors, including Toll-like receptors (TLRs) and C-type lectin receptors, recognize pathogen-associated molecular patterns (PAMPs), such as exotoxins, endotoxins, lipids, and microbial nucleic acids, as well as host-derived damage-associated molecular patterns (DAMPs). Engagement of PAMPs and DAMPs with PRRs initiates signaling cascades that culminate in the secretion of pro- and anti-inflammatory cytokines and chemokines, expression of cell adhesion molecules, and activation of adaptive immune responses [2,6]. Dysregulation of the balance between pro-inflammatory and anti-inflammatory signaling is a central feature of sepsis pathogenesis. In macrophages, these responses are influenced by lineage-specific activation states. M1 macrophages, stimulated by lipopolysaccharide (LPS) and interferon-γ (IFN-γ), predominantly produce pro-inflammatory cytokines and chemokines, whereas M2 macrophages, induced by IL-4 and IL-13, secrete anti-inflammatory mediators.

Activation of nuclear factor-kappa B (NF-κB) signaling following PAMP–PRR or DAMP–PRR interactions promotes the early production of cytokines, including interleukin 1 (IL-1), interleukin 6 (IL-6), interleukin 8 (IL-8), interleukin 12 (IL-12), IFN-γ, and TNF-α [6]. These responses further activate complement and coagulation pathways and enhance phagocytic activity. In addition to NF-κB, activator protein-1 (AP-1) regulates the expression of endothelial activation markers and inflammatory genes, including IL-6 and TNF-α [11]. IL-6 signaling activates Janus kinase (JAK)-mediated phosphorylation of signal transducer and activator of transcription 3 (STAT3), which contributes to the amplification and maintenance of systemic inflammation. Mice lacking STAT3 in neutrophils and macrophages exhibit exaggerated inflammatory responses and increased mortality [12]. Subsequent activation of neutrophils, B cells, and myeloid-derived suppressor cells promotes antibody production, including IgM and IgG. Conversely, anti-inflammatory responses contribute to immunosuppression by reducing Human Leukocyte Antigen—DR isotype (HLA-DR: MHC class II) expression, suppressing T-cell activity, and inducing immune cell apoptosis. This immunosuppressive state increases susceptibility to secondary opportunistic infections and contributes to sepsis-associated mortality [6,10,13].

## 3. Empty Hollow Nanoparticles as Antimicrobial Agents in Treatment of Sepsis

Sepsis progresses through three major clinical stages. The first stage, termed sepsis, is characterized by a systemic inflammatory response to infection involving both innate and adaptive immune mechanisms. Traditionally, sepsis has been identified by the presence of at least two of the following criteria: body temperature above 100.4 °F or below 96.8 °F, heart rate greater than 90 beats per minute, respiratory rate exceeding 20 breaths per minute, and a white blood cell count greater than 12,000/mm^3^ or less than 4000/mm^3^. However, diagnosis remains challenging because more than 40% of patients with clinical sepsis have negative microbial cultures [14]. This limitation complicates pathogen-directed therapy and may delay optimization of antimicrobial treatment. Regardless of the causative pathogen, sepsis is characterized by excessive activation of innate immune pathways, overproduction of inflammatory mediators, oxidative stress, endothelial dysfunction, and subsequent immune suppression. These pathological processes occur in both culture-positive and culture-negative sepsis. Biomarkers such as procalcitonin and C-reactive protein are frequently used to support diagnosis [15]. Early recognition and prompt initiation of antibiotics, fluid resuscitation, and source control measures, such as drainage of infected sites or amputation of necrotic tissue, are critical for improving patient outcomes.

If the infection and inflammatory response are not adequately controlled, sepsis may progress to severe sepsis, which is characterized by the development of organ dysfunction. Major organs, including the lungs, kidneys, heart, liver, and brain, may become impaired. Concurrent failure of two or more organ systems is referred to as MODS. Although MODS is a major contributor to sepsis-associated mortality, its precise pathophysiology remains incompletely understood, and therapeutic strategies aimed at reversing the condition, including anti-inflammatory agents and antioxidant enzymes, have demonstrated limited clinical efficacy [16]. Consequently, management of MODS remains largely supportive and focuses on preserving organ function. Mechanical ventilation is frequently required to manage respiratory failure; however, prolonged positive-pressure ventilation may contribute to ventilator-induced lung injury [17]. Similarly, renal replacement therapy is used to support kidney function but may be associated with complications such as hypotension, arrhythmias, circuit clotting, infection, and loss of nutrients and medications.

Persistent hypotension despite adequate fluid resuscitation with crystalloids or albumin marks progression to septic shock, the most severe stage of the disease. Administration of large volumes of fluid may contribute to fluid overload and further exacerbate organ dysfunction [18]. At this stage, organ failure becomes more severe, and the prognosis deteriorates substantially. Vasopressor agents are administered to maintain adequate blood pressure, while inotropic agents may be used to improve cardiac output [18]. However, these therapies are associated with significant adverse effects, including cardiac arrhythmias, impaired tissue perfusion, ischemic injury requiring limb amputation, worsening organ dysfunction, and thrombotic complications. In parallel, many patients develop profound immunosuppression, which compromises pathogen clearance and increases susceptibility to secondary and opportunistic infections. Mortality from septic shock increases with delays in antibiotic administration, and the emergence of antimicrobial resistance has further complicated sepsis management [13]. Indiscriminate antibiotic use has accelerated the development of antimicrobial resistance, thereby reducing treatment efficacy. Furthermore, failure to identify the causative microorganism may result in inappropriate antibiotic selection, which can promote the emergence and spread of resistant pathogens [19].

Although nanoparticles were initially developed primarily as inert drug-delivery vehicles, accumulating evidence indicates that they can directly influence immune responses independent of any encapsulated payload. Cargo-free nanoparticles are readily internalized by monocytes and macrophages, the key cellular mediators of sepsis pathophysiology, enabling direct interaction with innate immune signaling pathways and modulation of inflammatory responses [20]. In recent years, considerable efforts have been directed toward improving antimicrobial therapy through the development of empty nanoparticles, some of which possess intrinsic antimicrobial activity. These particles, commonly referred to as hollow nanoparticles, contain a hollow core that is not utilized for therapeutic cargo delivery. Hollow nanoparticles have attracted significant interest because of their unique catalytic and electrochemical properties [21]. In addition, nanoparticles may enhance antibiotic delivery to pathogens and prolong antibiotic half-life, thereby improving therapeutic efficacy [22].

Hollow nanoparticles can be fabricated using a variety of approaches, including hard-template, soft-template, self-templating, emulsion-assisted, and biomimetic techniques [23,24,25]. For instance, hollow mesoporous silica nanoparticles are commonly produced through emulsion-assisted methods, whereas biomimetic nanoparticles are often generated by coating nanoparticle templates with biologically derived membranes [24,25]. The choice of fabrication strategy influences key physicochemical properties, including particle size, shell thickness, surface chemistry, and loading capacity, which ultimately determine biological performance and therapeutic efficacy. Comprehensive physicochemical characterization is essential for evaluating hollow nanoparticle systems. Scanning electron microscopy (SEM) and transmission electron microscopy (TEM) are routinely used to confirm particle morphology and hollow architecture. Dynamic light scattering (DLS) is commonly employed to determine particle size distribution, polydispersity, and zeta potential, providing information on colloidal stability and surface charge. In addition, Fourier-transform infrared spectroscopy (FTIR), X-ray diffraction (XRD), and X-ray photoelectron spectroscopy (XPS) are frequently utilized to assess chemical composition, crystallinity, and surface functionalization [26,27,28,29,30].

Hollow metal nanoparticles (e.g., silver, gold, and zinc) and metal oxide nanoparticles (e.g., iron oxide, copper oxide, zinc oxide, and titanium dioxide) exhibit broad-spectrum antimicrobial activity, including effectiveness against MDR pathogens. Their antimicrobial effects are primarily mediated by the release of metal ions, which impair microbial viability through multiple mechanisms. These include disruption of cell membrane integrity, induction of ROS-mediated oxidative stress, interference with DNA replication and transcription, inhibition of protein synthesis and function, and suppression of bacterial biofilm formation [31,32,33,34,35]. Among these materials, silver nanoparticles have received considerable attention because of their antibacterial, antifungal, antimycobacterial, and antiviral activities, which are largely attributed to the release of silver ions [34,36]. In bacteria, silver ions electrostatically interact with sulfur-containing proteins present in the cell wall and cytoplasmic membrane, resulting in increased membrane permeability and enhanced intracellular uptake of silver ions. Once internalized, silver ions can inactivate respiratory enzymes, suppress ATP production, induce ROS generation, bind to DNA, and inhibit both DNA replication and protein synthesis, ultimately leading to microbial cell death [37,38]. The antimicrobial activity of silver nanoparticles has also been demonstrated against fungal pathogens. In *Candida albicans*, ROS generated by biogenic silver nanoparticles disrupt membrane integrity, induce oxidative stress, and promote cell death [39]. Furthermore, silver nanoparticles synthesized using green technologies have shown efficacy against several clinically relevant human mycopathogens [40,41]. Similarly, hollow TiO_2_ nanoparticles doped with silver ions exert antifungal activity against *Fusarium solani* and *Venturia inaequalis* through the formation of Ag–S and disulfide bonds, resulting in cellular damage and growth inhibition [42].

Iron oxide hollow nanoparticles have demonstrated considerable antibacterial activity against both Gram-positive and Gram-negative bacteria [43]. Because iron is an essential element involved in numerous physiological processes, iron oxide nanoparticles generally exhibit favorable biocompatibility in mammalian systems. However, exposure to excessive concentrations may disrupt iron homeostasis, resulting in iron accumulation and increased generation of reactive oxygen species (ROS), which can promote oxidative stress and cellular injury. Consequently, careful dose optimization and comprehensive toxicological evaluation are essential prerequisites for the safe clinical translation of these nanoparticles [43]. Several studies have investigated the antibacterial efficacy of iron oxide nanoparticles. Green-synthesized iron oxide nanoparticles have been reported to exhibit lower antimicrobial activity than streptomycin when administered alone. Nevertheless, co-administration with gentamicin produces larger zones of bacterial growth inhibition than treatment with gentamicin alone, suggesting a synergistic interaction and enhancement of antibiotic efficacy [43,44]. Although these findings are encouraging, direct quantitative comparisons with standard antimicrobial agents would provide a more robust assessment of the therapeutic potential of hollow iron oxide nanoparticles.

In addition to metal-based systems, hollow carbon-based nanoparticles, including fullerenes, graphene, and carbon nanotubes, possess intrinsic antimicrobial properties. These nanoparticles can impair microbial survival by disrupting cell membrane integrity, interfering with cellular metabolism, and inducing ROS-mediated oxidative stress [45,46,47]. However, the antimicrobial efficacy of carbon-based nanoparticles varies according to their physicochemical characteristics. For example, fullerenes exhibit greater activity against Gram-positive bacteria, likely because of enhanced membrane penetration. While this property may be advantageous in certain infections, it could limit efficacy in polymicrobial sepsis, where Gram-negative pathogens are frequently present [48]. In contrast, the antibacterial potential of graphene is often reduced by its tendency to agglomerate in aqueous environments [48]. This limitation may be mitigated through engineering approaches such as surface functionalization, polymer coating, and other modifications designed to improve nanoparticle dispersion and stability. Nevertheless, additional studies are required to optimize these strategies and determine their suitability for systemic therapeutic applications.

In chitosan-based hollow nanoparticles, positively charged glucosamine residues located on the nanoparticle surface interact electrostatically with negatively charged components of the bacterial cell membrane. These interactions disrupt cell wall integrity, leading to increased membrane permeability, osmotic imbalance, leakage of intracellular contents, and ultimately cell death. Chitosan exerts its antimicrobial effects primarily through electrostatic interactions that alter membrane permeability, followed by interference with DNA replication and subsequent bacterial lysis [49]. Through these mechanisms, hollow nanoparticles can target bacterial, fungal, and viral pathogens, thereby potentially intervening during the early stages of sepsis.

Despite their antimicrobial potential, the safety of these nanoparticles for systemic administration remains a significant concern. Although their antibacterial activity is attributed to preferential interactions with negatively charged bacterial membrane components, including LPS and lipoteichoic acids, nonspecific interactions with host cells and circulating blood components may also occur. Following systemic administration, nanoparticles can accumulate in major organs, including the liver, spleen, lungs, kidneys, and brain, where they may induce toxic effects. Some nanoparticles can cross the blood–brain barrier and promote neuroinflammatory responses [50]. In addition, uptake by the reticuloendothelial system can trigger inflammatory reactions and alter normal immune function [51]. The cationic surface charge of chitosan nanoparticles may further enhance interactions with endothelial cells and erythrocytes through electrostatic attraction, increasing cellular uptake and potentially contributing to endothelial injury, hemolysis, and cytotoxicity. Toxicity concerns are not limited to systemic administration, as adverse effects on host cells have also been reported following local application to wounds. Owing to these safety limitations, particularly those associated with metal- and chitosan-based nanoparticles, clinical translation has remained challenging.

Although hollow inorganic nanoparticles possess intrinsic antimicrobial activity, concerns regarding toxicity continue to restrict their development for the treatment of septic shock. For example, silver-containing nanoparticles release silver ions that can exert off-target effects on host tissues by disrupting cellular membranes and promoting ROS generation. Because patients with septic shock frequently present with pre-existing multi-organ dysfunction, additional toxicity associated with metal ion release may further exacerbate tissue injury and impair organ function. Consequently, metal ion-containing hollow nanoparticles, in their current form, may have limited suitability for the treatment of septic shock and require substantial optimization to improve their safety profile before clinical application.

As a result, recent research has increasingly focused on the immunomodulatory and anti-inflammatory properties of hollow nanoparticles rather than their direct antimicrobial effects. This approach may provide therapeutic benefit even in culture-negative sepsis or infections caused by antibiotic-resistant pathogens. Accordingly, the development of hollow nanoparticles is increasingly directed toward the modulation of dysregulated host immune responses rather than targeting specific microorganisms. Several hollow nanoparticle platforms have been engineered to exert therapeutic effects through scavenging ROS, adsorbing endotoxins and inflammatory mediators, modulating macrophage polarization, and suppressing excessive cytokine production. Because these mechanisms target host–pathogen interactions rather than individual pathogens, their administration does not require prior identification of the causative microorganism or knowledge of its antimicrobial resistance profile. Instead, hollow nanoparticles may serve as adjunctive host-directed therapies that restore immune homeostasis while conventional diagnostic investigations and antimicrobial treatments are ongoing.

## 4. Hollow Nanoparticles Not Containing Metal or Chitosan as Drivers of Immune Modulation in Sepsis

An effective nanoparticle-based therapy for septic shock should not only control microbial burden but also address the profound immune dysregulation that drives disease progression. Equally important, such therapeutic platforms must demonstrate an acceptable safety profile. Early studies suggested that empty hollow nanoparticles, particularly lipid-based formulations, could activate immune responses and promote pro-inflammatory signaling, raising concerns regarding their suitability for the treatment of sepsis. Excessive cytokine production contributes to the development of SIRS, thereby accelerating progression to septic shock and multi-organ dysfunction. Consistent with this concept, preclinical studies have shown that administration of ionizable lipid-containing empty lipid nanoparticles (eLNPs) induces robust IL-6 production in murine models [52,53]. Given that IL-6 is an established biomarker of sepsis severity, these findings are of particular concern. Clinically, elevated circulating IL-6 levels are strongly associated with disease severity, organ dysfunction, and poor outcomes in septic patients [54,55]. Consequently, conventional ionizable lipid-containing hollow lipid nanoparticles have generally been regarded as pro-inflammatory and therefore of limited therapeutic value for septic shock.

Recent advances in nanomaterial engineering have challenged this view by demonstrating that nanoparticle-induced immune responses are highly context-dependent and can be modulated through rational design. Several physicochemical properties, including particle size, shape, surface charge, porosity, and surface chemistry, play critical roles in determining immune recognition and downstream signaling pathways. Strategic optimization of these characteristics can substantially reduce, or even eliminate, the pro-inflammatory effects traditionally associated with hollow nanoparticle systems. Through careful engineering, nanoparticles that would otherwise promote immune activation can be redesigned to exert immunoregulatory or immunosuppressive effects, thereby attenuating excessive inflammation while minimizing unintended immune stimulation.

In addition to modulating cytokine production, engineered hollow nanoparticles can influence innate immune signaling pathways and regulate monocyte and macrophage polarization [53,54]. These effects may alter the balance between pro-inflammatory and anti-inflammatory immune responses and potentially contribute to the transition toward the immunosuppressive state that characterizes late-stage sepsis. Because this phase is associated with increased susceptibility to secondary infections and adverse clinical outcomes, the immunological consequences of nanoparticle therapy must be carefully evaluated. Accordingly, nanoparticle-induced immune activation and immune suppression should be viewed as interconnected and temporally dynamic processes rather than mutually exclusive outcomes.

**Figure 1:**
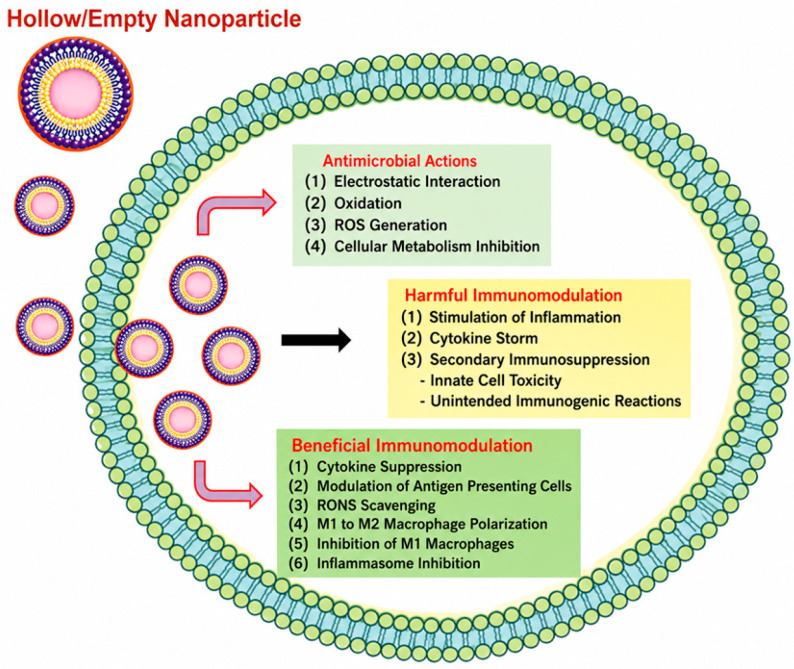
Functional effects of hollow/empty nanoparticles. Nanoparticles exert antimicrobial activity through membrane interaction and oxidative mechanisms and modulate immune responses, resulting in either pro-inflammatory toxicity or protective effects including cytokine suppression, reactive oxygen and nitrogen species (RONS) scavenging, macrophage M2 polarization, and inflammasome inhibition.

Based on the current literature, hollow nanoparticle platforms may be broadly categorized according to their reported immunomodulatory potential. Among the available systems, lipid-based nanoparticles appear to exhibit the most pronounced immunomodulatory effects, followed by polymeric, protein-based, biomimetic, inorganic hollow, hybrid, and carbon-based nanoparticles (Figure 1). However, this classification should not be considered a definitive ranking of therapeutic efficacy. Direct comparative studies evaluating these nanoparticle classes under identical sepsis conditions remain limited, and variations in nanoparticle composition, size, surface chemistry, dosage, route of administration, and experimental design complicate meaningful comparisons across studies. Consequently, this hierarchy represents a qualitative assessment of the currently available evidence and should be interpreted with caution. Well-controlled comparative studies using standardized sepsis models will be necessary to accurately determine the relative immunomodulatory efficacy of different hollow nanoparticle platforms.

Importantly, the biological activity of nanoparticles is strongly influenced by their physicochemical properties and formulation characteristics. As a result, nanoparticles belonging to the same class may induce either immunostimulatory or immunosuppressive responses depending on their design. This variability underscores the importance of rational nanoparticle engineering to maximize beneficial immune modulation while minimizing unintended inflammatory effects. Despite these challenges, hollow nanoparticles remain promising therapeutic candidates for septic shock because of their capacity to modulate dysregulated immune responses, suppress excessive inflammation, and potentially improve clinical outcomes beyond those achievable with conventional antimicrobial therapies alone.

### 4.1. Harmful Immunomodulatory Effects of Hollow Nanoparticles for Sepsis

**Figure 2:**
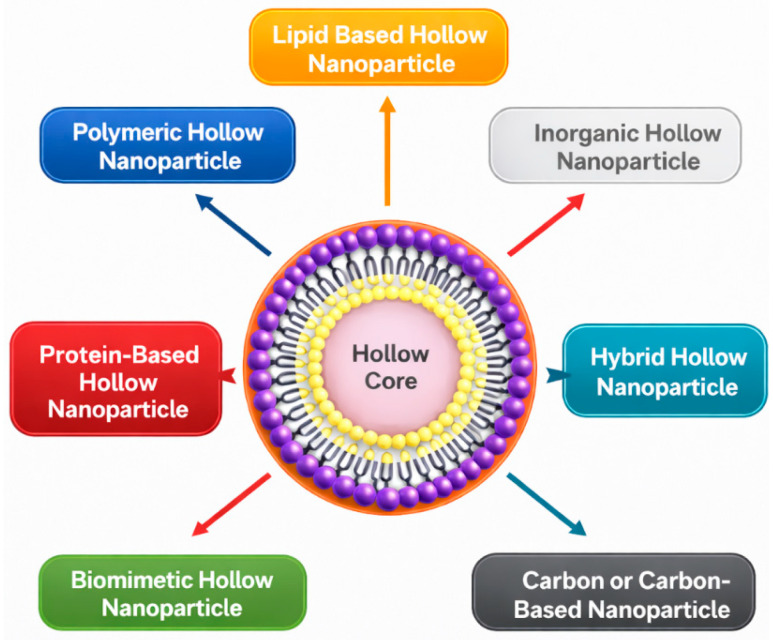
Potential qualitative classification of immunomodulatory hollow nanoparticles. Schematic showing major classes of immunomodulatory hollow nanoparticles based on composition, including lipid-based, polymeric, protein-based, biomimetic, inorganic, carbon-based, and hybrid nanoparticles.

Nanoparticles interact with the immune system through complex mechanisms that can result in either immune activation or immune suppression. Historically, hollow nanoparticles, particularly eLNPs, have been associated with immunostimulatory and pro-inflammatory effects, which have limited their perceived therapeutic utility in inflammatory disorders such as sepsis (Figure 2). This is particularly important in septic shock, where immune responses are highly heterogeneous and patients may exhibit either persistent hyperinflammation or profound immunosuppression. In individuals with ongoing hyperinflammation, further stimulation of inflammatory cytokine production is undesirable because it may exacerbate tissue injury and accelerate the development of multi-organ dysfunction. Conversely, in immunosuppressed patients, depletion and functional impairment of immune cells may reduce the capacity of nanoparticles to induce meaningful cytokine responses, thereby limiting their therapeutic efficacy. Consequently, the pro-inflammatory properties associated with early-generation empty lipid nanoparticles have restricted their potential application in the treatment of septic shock.

At the cellular level, ionizable lipid-based eLNPs stimulate the production of several pro-inflammatory cytokines, including IL-1, IL-6, IL-12, IFN-α, and IFN-γ, particularly in human monocyte-derived dendritic cells [20,52,53]. These responses are accompanied by increased expression of co-stimulatory molecules, including CD40 and OX40 ligand (OX40L), on dendritic cell populations within peripheral blood mononuclear cells, indicating enhanced activation of antigen-presenting cells. Collectively, these observations are characteristic of innate immune activation and have contributed to the perception that empty nanoparticles are inherently pro-inflammatory. However, growing evidence suggests that nanoparticle-induced immune activation is not a fixed response and may evolve into secondary immunosuppressive or tolerogenic programs depending on nanoparticle size, composition, surface charge, and the surrounding signaling environment.

The role of IL-6 in sepsis is particularly complex because this cytokine possesses both pro-inflammatory and anti-inflammatory functions. Nevertheless, elevated circulating IL-6 concentrations are consistently associated with increased disease severity, organ dysfunction, and poor clinical outcomes in patients with septic shock [56,57]. Consequently, induction of IL-6 by empty lipid nanoparticles may be detrimental during the early hyperinflammatory phase of sepsis, where additional cytokine production could intensify inflammatory injury and worsen organ damage. In contrast, the biological and therapeutic significance of eLNP-induced IL-6 production during the immunosuppressive phase of sepsis remains poorly understood. Therefore, despite the pleiotropic nature of IL-6 signaling, nonspecific induction of IL-6 by eLNPs may limit the clinical suitability of these nanoparticles for the treatment of septic shock.

Mechanistically, positively charged liposomes, including C14-amine-based formulations, can partially mimic the biological effects of LPS through activation of TLR4. Engagement of TLR4 activates both MyD88-dependent NF-κB and JNK signaling pathways as well as TRAM/TRIF-mediated signaling cascades, resulting in the production of pro-inflammatory cytokines and immunoregulatory mediators such as transforming growth factor-β (TGF-β) [58,59]. The induction of TGF-β represents a critical transition point at which nanoparticle-driven inflammatory responses may evolve into immunosuppressive programs [60]. Elevated TGF-β levels promote the development of tolerogenic dendritic cells, impair antigen presentation, and suppress effector T-cell responses, thereby contributing to immune regulation [61].

In addition, exposure to eLNPs has been shown to alter innate immune signaling thresholds, regulate T follicular helper cell differentiation, and promote monocyte polarization toward regulatory phenotypes [20]. Collectively, these changes may shift immune responses away from effective pathogen clearance and toward immune paralysis, a defining feature of late-stage sepsis. Thus, although empty hollow nanoparticles initially activate innate immune pathways, sustained or dysregulated signaling, particularly through TLR-dependent mechanisms, may paradoxically initiate immunosuppressive cascades. This duality highlights the importance of nanoparticle physicochemical characteristics in determining immune outcomes and underscores the need to distinguish early inflammatory signaling events from downstream regulatory and suppressive responses when evaluating nanoparticle-based therapies for septic shock and other immune-mediated disorders.

Although nanoparticles have traditionally been investigated for drug delivery and immune stimulation, accumulating evidence indicates that hollow nanoparticles can also induce immunosuppressive effects through multiple interconnected mechanisms. In sepsis, where immune responses frequently transition from an early hyperinflammatory state to profound immune dysfunction, nanoparticle-mediated immunosuppression may substantially influence disease progression and therapeutic outcomes. One important mechanism underlying nanoparticle-induced immunosuppression is direct cytotoxicity toward innate immune cells. Empty nanoparticles, particularly cationic formulations, have been reported to exert toxic effects on macrophages and monocytes, impairing both cellular viability and immune function. This toxicity is largely attributed to the physicochemical properties of cationic liposomes, especially the structure of their hydrophilic head groups, which can disrupt cellular membranes and compromise intracellular homeostasis [62,63]. Loss or dysfunction of these critical innate immune cells may accelerate the transition from systemic inflammation to the immunosuppressive phase of sepsis, resulting in impaired pathogen clearance and increased susceptibility to secondary infections.

Recent advances in nanoparticle engineering have focused on improving biocompatibility through optimization of particle size, incorporation of lipid components with more favorable immunomodulatory profiles, and surface modification strategies designed to minimize undesirable membrane interactions. Although there is currently no evidence that these approaches eliminate nanoparticle-associated toxicity, they can substantially influence physicochemical properties and biological behavior. Consequently, rational nanoparticle design may facilitate the development of safer nanomedicine-based therapies for septic shock and other immune-mediated diseases.

Beyond direct cytotoxic effects, nanoparticle composition can also influence immune responses through unintended immunogenic interactions. Polyethylene glycol (PEG), a surface-modifying polymer commonly used to prolong nanoparticle circulation and reduce rapid clearance, represents a notable example. Although PEG was originally considered biologically inert, increasing evidence indicates that repeated exposure to PEGylated nanoparticles can induce the formation of anti-PEG antibodies in certain individuals [64,65,66]. The development of these antibodies may accelerate the clearance of subsequently administered PEGylated nanoparticles through opsonization and phagocytic uptake, thereby reducing bioavailability and therapeutic efficacy. In the setting of septic shock, this increased phagocytic burden may further contribute to immune dysfunction and cellular exhaustion. To address these limitations, several next-generation nanoparticle platforms have been developed without PEG coatings. For example, phospholipid nanoparticle formulations such as VBI-S, as well as many biomimetic nanoparticle systems, exhibit improved biocompatibility by avoiding PEGylation altogether. Biomimetic hollow nanoparticles, including erythrocyte-, platelet-, leukocyte-, and bacterial membrane-coated nanoparticles, utilize naturally occurring membrane proteins and surface antigens to evade immune recognition and prolong circulation time. As a result, these systems can achieve stealth properties without relying on PEG-mediated surface modification, potentially reducing immunogenicity while maintaining favorable pharmacokinetic characteristics.

### 4.2. Hollow Nanoparticles as Beneficial Modulators of Immune Cells in Sepsis

The immunomodulatory properties of hollow nanoparticles are increasingly being leveraged in therapeutic strategies for inflammatory disorders such as sepsis and septic shock, where dysregulated immune activation and oxidative stress drive organ dysfunction and mortality. Among the most promising systems are biomimetic nanosponges, hollow nanoparticles coated with biological membranes derived from platelets, red blood cells, neutrophils, or macrophages, as well as liposomal hollow nanoparticles. These platforms can neutralize endotoxins or pore-forming toxins, sequester pro-inflammatory mediators, and attenuate oxidative stress. The intrinsic biological functionality of the membrane coating enhances immune compatibility and facilitates targeted immunomodulation, closely recapitulating physiological regulatory mechanisms observed in vivo. Beyond their ability to reduce the secretion of pro-inflammatory cytokines, hollow nanoparticles exert broader immunoregulatory effects by neutralizing elevated levels of ROS that accumulate during septic inflammation. Excess ROS disrupts immune cell signaling, impairs mitochondrial function, and perpetuates inflammatory feedback loops. By alleviating oxidative stress, hollow nanoparticles help stabilize immune cell function and prevent ROS-mediated amplification of inflammatory responses.

In addition to redox regulation, hollow nanoparticles influence dendritic cell behavior, a key determinant of immune balance during sepsis. These nanostructures can alter dendritic cell maturation, antigen processing, and co-stimulatory signaling, thereby shaping downstream immune responses toward controlled activation or immune tolerance. Hollow nanoparticles also impact adaptive immunity by modulating T-cell activation, differentiation, and effector capacity, helping to counteract sepsis-associated T-cell dysfunction and immune exhaustion. Through simultaneous regulation of oxidative stress, innate immune signaling, and adaptive immune responses, hollow nanoparticles can function as multifunctional immunosuppressive platforms that contribute to the restoration of immune homeostasis and reduction in disease severity in sepsis.

#### 4.2.1. Scavenging of Reactive Oxygen and Nitrogen Species

The early hyperinflammatory phase of sepsis is characterized by excessive oxidative and nitrosative stress arising from the overproduction of ROS and reactive nitrogen species (RNS). These include superoxide anion (O_2_^−^), hydrogen peroxide (H_2_O_2_), hydroxyl radicals (•OH), singlet oxygen (^1^O_2_), nitric oxide (NO), nitrogen dioxide (NO_2_), and peroxynitrite (ONOO^−^). In addition to amplifying inflammatory signaling pathways, these reactive species disrupt mitochondrial bioenergetics, impair autophagic clearance, and activate apoptotic pathways, collectively contributing to cellular dysfunction and the development of multi-organ failure [67]. Dysregulated production of RONS also compromises immune homeostasis by aberrantly activating or suppressing immune cells, including dendritic cells, neutrophils, macrophages, and T lymphocytes. These alterations promote immune tolerance, sustain inflammation, and exacerbate tissue injury [68].

Among inorganic nanomaterials, hollow manganese dioxide (H-MnO_2_) nanoparticles have emerged as promising ROS-scavenging platforms in preclinical studies. These nanoparticles attenuate oxidative stress through redox-cycling mechanisms involving catalytic decomposition of hydrogen peroxide and the release of Mn^2+^ ions, thereby helping to restore redox balance within inflamed tissues. Owing to their antioxidant and immunomodulatory properties, H-MnO_2_ nanoparticles have been investigated in a variety of disease settings, including cancer, atopic dermatitis, osteoarthritis, and wound healing [69]. Despite these encouraging findings, degradation of H-MnO_2_ nanoparticles may lead to the release of manganese ions, raising concerns regarding tissue accumulation and off-target toxicity. This issue is particularly relevant in septic shock, where hepatic and renal dysfunction may impair metal clearance and increase susceptibility to adverse effects. Furthermore, most available evidence has been generated from preclinical models of diseases other than septic shock, limiting the ability to directly extrapolate these findings to septic shock. Therefore, comprehensive toxicological, pharmacokinetic, and biodistribution studies are required to determine whether the therapeutic benefits associated with oxidative stress reduction outweigh the potential risks of manganese exposure before these nanoparticles can be considered for clinical application in septic shock.

Similarly, mesoporous polydopamine nanoparticles (mPDA NPs) and other hollow polydopamine (PDA)-based nanostructures exhibit potent antioxidant activity and effectively attenuate oxidative stress. These properties are particularly relevant in sepsis, where ischemia–reperfusion injury contributes substantially to cellular and mitochondrial damage during the inflammatory phase of the disease. In addition to their antioxidant effects, PDA-based nanoparticles demonstrate favorable biocompatibility and minimal hemolytic activity while suppressing inflammatory responses through the reduction in pro-inflammatory cytokine production [70]. Despite these advantageous properties, concerns remain regarding the hemocompatibility of PDA nanoparticles. Studies have shown that PDA nanoparticles can promote platelet activation and adhesion and may interact with coagulation factors and components of the complement system, potentially disrupting normal hemostatic processes [71]. Because septic shock is frequently associated with coagulation abnormalities, including disseminated intravascular coagulation, such effects could further exacerbate underlying coagulopathy. Therefore, a comprehensive evaluation of the hemocompatibility, thrombogenic potential, and coagulation-related effects of PDA nanoparticles is essential before their clinical application in septic shock can be considered.

Collectively, these findings highlight the potential of RONS-scavenging hollow nanoparticles as immunomodulatory and cytoprotective platforms for mitigating oxidative stress-driven immune dysregulation in sepsis. By reducing excessive oxidative and nitrosative stress, these nanoparticles may attenuate inflammatory tissue injury, preserve cellular function, and limit the progression of organ damage. Nevertheless, these findings should be interpreted cautiously because most RONS-scavenging hollow nanoparticle systems remain at an early stage of preclinical development. Furthermore, studies are required to comprehensively assess their safety, biodistribution, pharmacokinetics, and long-term therapeutic efficacy before clinical translation for the treatment of septic shock can be considered.

#### 4.2.2. Immunosuppressive and Anti-Inflammatory Responses

Mesoporous polymeric glycine nanoparticles (GlyNPs) with diameters below 50 nm exhibit notable anti-inflammatory properties and have been investigated primarily in wound-healing applications [72]. Their small size facilitates efficient uptake by immune cells, particularly macrophages, enabling direct modulation of inflammatory signaling pathways. In LPS-stimulated macrophages, GlyNPs significantly reduce the production of several inflammatory mediators, including NO, interleukin-6 (IL-6), interleukin-10 (IL-10), TNF-α, nuclear factor-κB (NF-κB), and IFN-γ, demonstrating broad immunomodulatory activity [73]. The immunological consequences of GlyNP-mediated IL-10 suppression may depend on the stage of sepsis. Studies using cecal ligation and puncture (CLP) models have shown that IL-10 can exert either beneficial or detrimental effects depending on the phase of disease progression [74]. Consequently, suppression of IL-10 during the early hyperinflammatory stage could potentially exacerbate inflammation, whereas a reduction in excessive IL-10 during the immunosuppressive phase may help restore immune competence [75,76]. Therefore, the therapeutic efficacy of GlyNPs is likely to be highly dependent on treatment timing, and additional studies are required to determine their overall impact on immune regulation during septic shock. Therefore, the therapeutic efficacy of GlyNPs is likely to be highly dependent on treatment timing, and additional studies are required to determine their overall impact on immune regulation during septic shock.

Hybrid hollow cerium oxide (hCeO_2_) nanoparticles attenuate oxidative stress through ROS scavenging and suppress inflammation by inhibiting the cystatin B–NLRP3 (NOD-like receptor protein 3) inflammasome pathway [77]. Although inhibition of NLRP3 signaling may reduce tissue injury during sepsis, it could also compromise host defense mechanisms against infection. However, cerium oxide nanoparticles possess intrinsic antimicrobial activity against a broad spectrum of pathogens, which may help preserve pathogen control despite suppression of inflammasome activation [78]. Further investigation is required to determine whether these nanoparticles can effectively balance antimicrobial defense with control of excessive inflammation, particularly during the early stages of sepsis. Similarly, hollow manganese dioxide- and cerium oxide-based nanoparticles suppress NF-κB, AP-1, and IL-6 signaling through ROS scavenging and inhibition of mitogen-activated protein kinase (MAPK) pathways [79,80]. Despite their promising therapeutic potential, concerns regarding the toxicity and long-term safety of metal oxide-based nanoparticles remain unresolved. Consequently, comprehensive studies evaluating their biocompatibility, efficacy, biodistribution, and toxicological profiles are required before clinical translation for sepsis therapy can be considered.

More recently, biomimetic nanoparticles have emerged as advanced platforms for sepsis treatment because of their ability to simultaneously target inflammation, limit infection, and modulate immune cell function [81]. Surface functionalization with natural cell membranes derived from immune cells, as well as natural or synthetic vesicles, enhances their immunoregulatory properties, improves biocompatibility, and prolongs stability and retention within biological environments [81]. Biomimetic cell membrane-coated nanoparticles are typically composed of a synthetic nanoparticle core surrounded by a membrane shell derived from living cells, thereby combining the functional advantages of both components. One example is erythrocyte membrane-coated poly(lactic-co-glycolic acid) (PLGA) nanoparticles generated from erythrocyte-derived vesicles [82]. Among biomimetic systems developed for sepsis, macrophage membrane-coated nanoparticles incorporating metal–polyphenol antioxidant cores and the flavonoid quercetin have demonstrated potent antioxidative and anti-inflammatory properties. In cecal ligation and puncture models of sepsis, these nanoparticles significantly reduce disease severity by promoting macrophage repolarization toward an M2 phenotype, decreasing ROS accumulation, and restoring mitochondrial function [83]. Although these nanoparticles retain macrophage membrane-associated receptors capable of binding inflammatory mediators, they lack the intracellular signaling machinery necessary for receptor-mediated signal transduction. Consequently, ligand binding does not trigger the downstream inflammatory responses that occur in viable macrophages. Instead, these biomimetic nanoparticles function as decoys that sequester cytokines and other inflammatory mediators, thereby reducing their availability to circulating immune cells during septic shock. Through this mechanism, excessive systemic inflammation may be attenuated more rapidly, potentially limiting organ injury and improving recovery. Nevertheless, further studies are required to determine how receptor orientation, membrane integrity, and nanoparticle composition influence cytokine-binding efficiency, biological activity, and therapeutic efficacy.

Biomimetic nanoparticles have also demonstrated the ability to restore dendritic cell and T-cell functions that are frequently impaired during sepsis-associated immunosuppression. By recapitulating key structural and functional features of natural immune cell membranes, these nanoparticles facilitate physiologically relevant immune interactions that enhance antigen presentation and adaptive immune responses. For example, mesoporous silica nanoparticles coated with dendritic cell membranes retain functional surface molecules involved in T-cell activation, enabling efficient stimulation of CD8^+^ cytotoxic T lymphocytes through coordinated interactions involving PD-1, CD3, and CD28 signaling pathways between dendritic cells and T cells [84]. This membrane-mediated crosstalk promotes T-cell priming and cytotoxic differentiation, thereby helping to counteract the T-cell exhaustion commonly observed during sepsis.

Biomimetic gold nanoparticles coated with bacterial outer membrane vesicles (BM-AuNPs) represent another promising immunomodulatory platform. These nanoparticles mimic PAMPs while maintaining controlled immune activation. BM-AuNPs effectively stimulate dendritic cell maturation and antigen presentation, leading to enhanced activation of both T-cell and B-cell responses [85]. Through these mechanisms, biomimetic nanoparticles can bridge innate and adaptive immunity and may help restore immune competence without inducing excessive inflammation. Collectively, these findings highlight the potential of biomimetic nanoparticle platforms to reverse sepsis-associated immune dysfunction through selective enhancement of dendritic cell activity and adaptive immune responses.

Unlike inorganic metal-containing nanoparticles, which frequently suppress inflammation through direct inhibition of NF-κB signaling, biomimetic nanoparticles generally exert indirect anti-inflammatory effects through attenuation of TLR signaling, neutralization of microbial toxins, or sequestration of inflammatory cytokines. Inhibition of these upstream pathways ultimately reduces transcription of pro-inflammatory mediators, including IL-6, TNF-α, and IL-1β. In contrast, information regarding the effects of biomimetic and inorganic nanoparticles on AP-1 and STAT3 signaling during sepsis remains limited. Although direct evidence is currently lacking, membrane-camouflaged biomimetic nanoparticles have been reported to influence MAPK pathways, suggesting a potential indirect effect on AP-1 activity [81]. Importantly, patient-specific factors, including age, comorbidities, and genetic variability, may influence TLR signaling and other immune pathways, thereby affecting the therapeutic efficacy of hollow nanoparticles in septic shock. Future studies employing clinically relevant preclinical models are required to determine how these factors influence treatment responses and to identify patient populations most likely to benefit from nanoparticle-based therapies.

Although several nanoparticle formulations have been associated with pro-inflammatory effects, certain nanoparticles exhibit anti-inflammatory and immunomodulatory properties. For example, hollow silver nanoparticles approximately 100 nm in diameter have been shown to promote macrophage polarization toward the anti-inflammatory M2 phenotype. Similarly, silver–polyvinylpyrrolidone (Ag–PVP) nanoparticles attenuate inflammatory signaling and reduce inflammation in experimental models of *Chlamydia* infection in burn wounds [86,87,88,89,90]. Because M2 macrophage polarization is considered a promising strategy for limiting excessive inflammation during sepsis, these immunomodulatory effects may have therapeutic relevance. However, concerns remain regarding the intrinsic toxicity of metal-containing nanoparticles, particularly because of metal ion release and the potential induction of oxidative stress. It remains unclear whether coating metal nanoparticle cores with natural cell membranes can eliminate metal-associated toxicity. Additionally, the safety of administering metal-containing nanoparticles into body cavities or the systemic circulation requires careful evaluation, as nanoparticle accumulation, metal ion release, or local tissue reactions could potentially exacerbate inflammatory injury. Therefore, comprehensive investigations of the biodistribution, toxicity, and long-term safety of biomimetic metal-containing nanoparticles are necessary before their clinical application in septic shock can be considered.

Because silver-based hollow nanoparticles possess intrinsic antimicrobial activity mediated through the release of silver ions, distinguishing their direct antimicrobial effects from their immunomodulatory actions on macrophages remains challenging. Although these nanoparticles have been reported to increase the expression of M2-associated markers and attenuate inflammatory responses, it is unclear whether such phenotypic changes preserve, enhance, or impair essential antimicrobial functions. In particular, the effects of nanoparticle-induced macrophage polarization on phagocytosis, microbial killing, and pathogen clearance have not been thoroughly investigated. Likewise, although inorganic hollow nanoparticles, including manganese dioxide-, cerium oxide-, and silver-based nanostructures, have been associated with the development of anti-inflammatory macrophage phenotypes, direct evidence demonstrating active reprogramming of M1 macrophages into M2 macrophages remains limited. Therefore, future studies should determine whether hollow nanoparticles can preserve critical host-defense mechanisms while simultaneously exerting beneficial immunomodulatory effects.

Excessive pulmonary edema and uncontrolled inflammatory responses during bacterial sepsis frequently contribute to the development of acute lung injury (ALI), which may subsequently progress to acute respiratory distress syndrome (ARDS). In recent years, hollow nanoparticles have emerged as promising therapeutic platforms for the treatment of ALI because of their favorable physicochemical characteristics and immunomodulatory properties. For example, intratracheal administration of ultrasmall molybdenum nanodots (MNDs) with high biocompatibility has been shown to markedly reduce oxidative stress and pulmonary inflammation, thereby promoting recovery from sepsis-associated lung injury. Mechanistic studies have demonstrated that these nanoparticles possess potent ROS-scavenging activity and modulate inflammatory cell death pathways, including pyroptosis mediated through activation of the NLPR3 inflammasome [90].

More recently, hollow manganese silicate (MS) nanoparticles have been developed as a targeted therapeutic strategy for *Pseudomonas aeruginosa*-induced ALI. These nanoparticles effectively neutralize ROS while simultaneously modulating host immune responses, thereby reducing pulmonary tissue injury. In addition, MS nanoparticles function as efficient pulmonary drug delivery systems, enabling targeted antibiotic transport to the site of infection and enhancing antimicrobial efficacy through synergistic mechanisms [91]. Because excessive ROS generation and amplified pro-inflammatory signaling are major contributors to the development and progression of ARDS during septic shock, therapeutic strategies aimed at restoring redox homeostasis and regulating immune responses are of considerable interest [92]. Collectively, these findings highlight the potential of hollow nanoparticle-based therapies as promising approaches for the treatment of sepsis-induced ALI and ARDS. Nevertheless, additional studies are required to establish their long-term safety, therapeutic efficacy, and translational potential in clinically relevant models of septic shock.

#### 4.2.3. Neutralization of Bacterial Products

Beyond approaches aimed at reducing pathogen burden and suppressing excessive inflammatory responses, an important therapeutic objective in septic shock is the neutralization of bacterial endotoxins, exotoxins, and other microbial products that drive systemic inflammation. Consequently, increasing attention has been directed toward hollow biomimetic nanoparticles as therapeutic platforms for sepsis because of their intrinsic ability to sequester and neutralize bacterial toxins. Nanoparticles coated with neutrophil or macrophage membranes have demonstrated efficient binding and neutralization of bacterial endotoxins and other pro-inflammatory mediators without inducing additional immune activation [56].

Similarly, biomimetic nanoparticles and nanosponge formulations coated with red blood cell (RBC) membranes have been shown to substantially reduce toxin-induced cytotoxicity while retaining the ability to adsorb bacterial products [81,93,94]. BC membrane-coated nanosponges are particularly effective at scavenging pore-forming toxins that would otherwise disrupt host cell membranes, thereby diverting these virulence factors away from their physiological targets. In vivo studies have demonstrated that such nanosponges efficiently bind staphylococcal α-hemolysin following subcutaneous toxin challenge in murine models [92]. In addition to toxin sequestration, RBC membrane-coated nanosponges indirectly suppress NF-κB signaling by reducing the availability of circulating toxins [92]. Despite these promising findings, several important questions remain unresolved. Existing studies have primarily focused on toxin binding and therapeutic effects in toxin-challenged animal models and have not quantified the maximum toxin-binding capacity of individual nanoparticles or determined the number of toxin molecules that can be sequestered before saturation. Furthermore, previous studies indicate that RBC membrane-coated nanoparticles are predominantly cleared by the mononuclear phagocyte system, particularly in the liver and spleen [95,96]. However, the biological fate of toxin-bound nanosponges following sequestration remains poorly understood. Consequently, quantitative estimates of toxin occupancy and the in vivo clearance kinetics of toxin–nanosponge complexes cannot currently be determined from available data.

Further advances in this field include RBC membrane-coated nanogels crosslinked through disulfide bonds, which effectively neutralize pore-forming toxins produced by methicillin-resistant *Staphylococcus aureus* (MRSA). Following toxin sequestration, these nanoparticles are readily internalized by macrophages, where the disulfide crosslinkers are cleaved within the reducing endosomal environment. This process enables controlled degradation of the nanogel matrix and provides a redox-responsive platform for intracellular antibiotic release when these hollow nanoparticles are used as drug delivery vehicles [97]. Consistent with their broad detoxification capabilities, RBC membrane-coated polymeric nanosponges have been shown to bind multiple pore-forming toxins, including α-hemolysin from MRSA, listeriolysin O from *Listeria monocytogenes*, and streptolysin O from Group A *Streptococcus*, highlighting their potential as versatile platforms for neutralizing diverse bacterial virulence factors [85].

Biomimetic nanoparticles coated with macrophage plasma membranes provide a distinct therapeutic advantage because they can sequester pro-inflammatory cytokines without triggering downstream inflammatory signaling. Because the early hyperinflammatory phase of sepsis is largely initiated by endotoxin release, particularly LPS from Gram-negative bacteria, targeted neutralization of LPS represents an attractive therapeutic strategy. Although polymyxin antibiotics effectively bind and neutralize endotoxins, their clinical utility is limited by dose-dependent nephrotoxicity and neurotoxicity. Consequently, the development of biocompatible hollow nanoparticles with intrinsic endotoxin-neutralizing capabilities has emerged as a promising alternative approach for the treatment of sepsis and septic shock. In this context, macrophage membrane-coated polymeric nanoparticles have been engineered to mimic the endotoxin-binding properties of native macrophages. These biomimetic nanoparticles efficiently bind LPS in murine models of *Escherichia coli* infection while simultaneously functioning as cytokine decoys that intercept inflammatory mediators and attenuate propagation of the septic inflammatory response [98]. Through sequestration of both LPS and pro-inflammatory cytokines, macrophage membrane-coated nanoparticles reduce activation of NF-κB-dependent inflammatory pathways and thereby suppress excessive inflammation [98]. These findings highlight the potential of biomimetic hollow nanoparticles as multifunctional therapeutic platforms capable of simultaneously neutralizing microbial toxins and modulating dysregulated host immune responses during septic shock.

Platelet activation is a hallmark of sepsis, and the development of thrombocytopenia is strongly associated with increased disease severity and mortality, highlighting the critical role of platelets in maintaining immune and inflammatory homeostasis. Beyond their established function in hemostasis, platelets also participate directly in innate immune defense, including protective responses against *Staphylococcus aureus* infection. Leveraging these biological properties, biodegradable polymeric nanoparticle cores have been functionalized with platelet-derived membranes to generate biomimetic platelet nanoparticles. These platelet membrane-coated nanoparticles efficiently sequester *S. aureus*-derived toxins, thereby reducing toxin-induced injury to macrophages and other host cells. *In vitro* studies have demonstrated that platelet membrane-coated nanoparticles attenuate both macrophage- and platelet-mediated toxicity induced by MDR-resistant *S. aureus*. By protecting immune cells from toxin-mediated damage and preserving their functional capacity, these nanoparticles may enhance host antimicrobial defenses and contribute to improved bacterial clearance [99]. However, the precise mechanisms responsible for the observed reduction in bacterial burden remain incompletely understood and warrant further investigation. Supporting the therapeutic potential of platelet membrane-based biomimetic systems, platelet membrane-coated copper silicate hollow microspheres have also been shown to adsorb LPS and suppress inflammatory responses during wound healing, further highlighting the intrinsic anti-inflammatory properties of platelet membranes [100].

Although no hollow nanoparticle-based therapy has yet completed clinical trials for the treatment of sepsis, the broad mechanistic versatility of these platforms makes them promising candidates for septic shock therapy. Early-generation lipid-based nanoparticles were frequently associated with excessive immune activation and dysregulated inflammatory responses, limiting their applicability in sepsis. However, advances in nanoparticle engineering have demonstrated that interactions between lipid nanoparticles and the immune system can be precisely controlled through rational modification of physicochemical properties. Through careful design, hollow nanoparticles, particularly biomimetic nanoparticle systems, can be engineered to combine multiple therapeutic functions within a single platform. These functions include antimicrobial activity, ROS scavenging, neutralization of bacterial antigens and toxins (including endotoxins and pore-forming virulence factors), and sequestration of inflammation-driving microbial products. In addition, these nanoparticles can modulate innate and adaptive immune responses, promoting controlled anti-inflammatory and immunoregulatory phenotypes. Collectively, such multifunctional hollow nanoparticle systems represent a promising antibiotic-independent therapeutic strategy for restoring immune homeostasis, limiting organ injury, and improving clinical outcomes in septic shock and its associated complications.

## 5. VBI-S: A Hollow Nanoparticle-Based Therapy in Clinical Trials for Septic Shock

The lack of FDA-approved therapeutics specifically indicated for preventing progression of severe infection to sepsis or for the treatment of septic shock has substantially limited therapeutic options and clinical outcomes. Among hollow nanotechnology-based therapeutics, VBI-S is currently the only agent that has undergone clinical evaluation for efficacy and safety in patients with septic shock-associated hypotension and multi-organ dysfunction. VBI-S, developed by Vivacelle Bio Inc. (Kansas City, MO, USA), is an intravenously administered phospholipid nanoparticle colloid that was evaluated in a multicenter, open-label single-arm Phase 2a pilot clinical study involving twenty patients with severe septic shock. Enrolled patients had Sequential Organ Failure Assessment (SOFA) scores of ≥8, including eleven patients with SOFA scores greater than 15, corresponding to a predicted mortality of approximately 90%. This cohort therefore represented a population with severe disease and a high risk of mortality (mean SOFA score: 14.0 ± 2.92) [101]. The efficacy and safety of VBI-S are currently being investigated in another ongoing randomized Phase 2b/3 trial comparing standard of care alone with standard of care plus VBI-S in patients with septic shock.

Administration of VBI-S in the Phase 2a trial was associated with rapid correction of refractory hypotension in patients who had failed conventional intravenous fluid resuscitation and remained dependent on vasopressor therapy. Before treatment, the mean arterial pressure (MAP) was 64.5 ± 4.27 mmHg despite standard supportive care. Following VBI-S infusion, MAP increased to 77.5 ± 4.73 mmHg, with all treated patients (100%) demonstrating an increase of at least 10 mmHg. The mean time for MAP improvement was 90.35 ± 101.40 min, with individual response times ranging from 15 to 378 min. The initial volume of VBI-S administered was 561.0 ± 372.3 mL, and the cumulative dose delivered over 48 h was 905.1 ± 352.0 mL. These volumes are substantially lower than those typically required during conventional fluid resuscitation for septic shock, which may contribute to fluid overload and exacerbate organ dysfunction [101,102].

Importantly, hemodynamic stabilization achieved following VBI-S administration was sustained despite progressive reduction in vasopressor support, and approximately 55% of patients were successfully weaned from vasopressors within 48 h. Beyond its hemodynamic effects, VBI-S treatment was associated with improvements in oxygenation and renal function, together with reduced hepatic dysfunction, findings consistent with enhanced tissue perfusion and attenuation of systemic inflammation. These physiological improvements translated into a reduction in overall organ dysfunction, as reflected by a decrease in mean SOFA score to 11.4 ± 3.858 within 24 h of treatment [101]. VBI-S was generally well tolerated, with reversible hyperlipidemia representing the only treatment-related adverse event. This effect was resolved spontaneously within one week of administration.

VBI-S is composed of a heterogeneous population of nanoscale liposomes and micelles formulated from egg lecithin, soybean oil, and glycerol, with a mean particle diameter of approximately 51 nm as determined by electron microscopy [103,104]. Its therapeutic effects in septic shock-associated hypotension and multi-organ dysfunction are hypothesized to be mediated, in part, through modulation of nitric oxide (NO) bioavailability. Because NO is highly lipophilic, it may preferentially partition into the hydrophobic lipid environment of VBI-S, facilitating sequestration of excess NO and its redistribution to regions with relative NO deficiency, thereby contributing to restoration of vascular homeostasis. Direct confirmation of this mechanism *in vivo* is challenging because NO has an extremely short half-life of approximately 2 milliseconds. However, *in vitro* mass spectrometry and chemiluminescence studies demonstrated greater NO uptake by VBI-S than by water and rapid NO release from the nanoparticles, supporting their potential role as dynamic NO carriers [101]. The observed increases in mean arterial pressure and improvements in pulmonary and renal function following VBI-S administration are consistent with this proposed mechanism.

These effects are unlikely to be explained solely by volume expansion, as patients were enrolled only after maximal conventional fluid resuscitation and were considered fluid unresponsive. In this setting, additional fluid administration is generally contraindicated because of the risk of worsening fluid overload and organ dysfunction. Nevertheless, VBI-S increased mean arterial pressure while improving organ function, suggesting a mechanism beyond simple volume replacement. Further studies are needed to directly evaluate the effects of VBI-S on NO distribution and bioavailability in vivo.

In addition to its effects on NO bioavailability, VBI-S may also contribute to improved tissue oxygenation. Similar to NO, oxygen is lipophilic and can partition into the lipid components of VBI-S. Consequently, the phospholipid colloid may function as an intravascular oxygen carrier, facilitating oxygen transport throughout the circulation [101]. However, the extent to which oxygen transport contributes to the observed hemodynamic and organ-protective effects of VBI-S remains unclear. Mechanistic studies are ongoing to determine the relative contribution of oxygen delivery compared with other proposed biological activities of VBI-S. Like VBI-S, another formulation, VBI-1, with a similar composition, has been found to be capable of hemodynamic improvements and reverse hemorrhagic shock in a rat clinical death model [103,104]. Therefore, beyond regulating NO homeostasis, VBI-S may promote hemodynamic stabilization and organ recovery through enhanced tissue oxygen delivery, a property that is enabled by its oxygen-carrying capacity and nanoscale architecture.

## 6. Regulatory and Translational Challenges of Hollow Nanoparticle-Based Therapies for Sepsis and Septic Shock

Hollow nanoparticles have attracted considerable attention as immunomodulatory nanotherapeutics for the treatment of sepsis and septic shock because of their ability to simultaneously target inflammation, oxidative stress, and microbial virulence factors. Although numerous preclinical studies have demonstrated promising therapeutic potential, significant manufacturing, regulatory, and safety-related challenges continue to impede their clinical translation [105].

One of the major barriers to clinical implementation is the scalable and reproducible manufacturing of hollow nanoparticles. Most hollow nanostructures are produced through multistep fabrication processes involving template synthesis, shell formation, and template removal, which can be difficult to standardize for large-scale production. This challenge is further amplified for biomimetic nanoparticles that incorporate cellular membranes or other biological components, introducing additional variability in composition and manufacturing. Successful clinical translation requires production under Good Manufacturing Practice (GMP) conditions with stringent control of critical quality attributes, including particle size distribution, morphology, surface chemistry, loading efficiency, and physicochemical stability. Even minor variations in these parameters may substantially influence biodistribution, pharmacokinetics, therapeutic efficacy, safety, and batch-to-batch reproducibility [106].

Formulation stability and sterility represent additional challenges for intravenously administered nanoparticle therapeutics. Because patients with septic shock are highly susceptible to infection and systemic inflammatory complications, nanoparticle formulations must remain sterile and endotoxin-free throughout manufacturing, storage, and administration. However, conventional sterilization techniques, including autoclaving, gamma irradiation, and membrane filtration, may alter nanoparticle architecture, surface properties, or biological activity. In addition, long-term storage may promote aggregation, oxidation, or structural degradation, potentially reducing therapeutic efficacy and increasing toxicity. Consequently, formulation-specific sterilization and storage strategies are required to preserve nanoparticle integrity and shelf-life [107].

Another important limitation is the absence of universally accepted standards for nanoparticle characterization. Regulatory evaluation requires a comprehensive assessment of parameters such as particle size, surface charge, shell thickness, porosity, composition, colloidal stability, and batch-to-batch consistency. However, the lack of standardized analytical methodologies specifically optimized for nanoparticle systems complicates product comparison, quality assessment, and regulatory review, thereby slowing clinical development and commercialization.

Safety remains a particularly critical concern in septic shock, where patients frequently present severe immune dysregulation and multi-organ dysfunction. Inorganic hollow nanoparticles containing metals or metallic components may release ions or generate ROS, potentially aggravating tissue injury and organ accumulation/damage. Therefore, extensive toxicological evaluation, including acute, chronic, reproductive, neurotoxicity, and genotoxicity studies, is required, especially for metallic hollow nanoparticles, before clinical application. Comprehensive investigation of biodistribution, biodegradation, clearance mechanisms, and long-term tissue retention is also essential to minimize the risk of off-target accumulation and delayed toxicity.

As hollow nanoparticles are specifically engineered to interact with immune pathways, unintended immunological effects represent an additional translational challenge. Nanoparticles may activate complement pathways, alter cytokine networks, stimulate antibody production, or induce unexpected immune responses that compromise both efficacy and safety. Excessive immunosuppression may also increase susceptibility to secondary infections during the immunosuppressive phase of sepsis. Consequently, a detailed investigation of nanoparticle–immune system interactions is necessary to establish an acceptable safety profile [108].

Hemocompatibility represents another important consideration for intravenously administered nanomedicines. Hollow nanoparticles may interact directly with erythrocytes, platelets, plasma proteins, and endothelial cells, potentially causing hemolysis, platelet activation, complement activation, coagulation abnormalities, or vascular injury. These effects are particularly concerning in patients with septic shock, who frequently exhibit endothelial dysfunction, coagulopathy, and microvascular abnormalities. Accordingly, comprehensive hemocompatibility assessments should be incorporated into preclinical development programs [108,109].

From a regulatory perspective, existing pharmaceutical evaluation frameworks are not always ideally suited to the unique properties of nanomedicines. The biological behavior of hollow nanoparticles is strongly influenced by nanoscale physicochemical characteristics that may not be adequately addressed by conventional regulatory approaches. Therefore, early engagement with regulatory agencies, implementation of standardized characterization methodologies, and development of nanomedicine-specific regulatory guidelines will be important for facilitating clinical translation [107].

Overall, successful clinical implementation of hollow nanoparticle-based therapies will require robust manufacturing processes, standardized quality-control procedures, optimized formulation stability, and comprehensive safety evaluation. In addition, well-designed clinical trials will be necessary to establish long-term safety, efficacy, and therapeutic benefit across the heterogeneous patient populations encountered in sepsis and septic shock. Continued advances in nanoparticle manufacturing, toxicological assessment, and regulatory science are expected to accelerate the development and clinical translation of next-generation hollow nanoparticle therapeutics.

## 7. Future Directions

Hollow nanoparticles represent a rapidly advancing class of immunomodulatory nanotherapeutics with significant potential for the treatment of septic shock. Despite encouraging preclinical findings, several scientific, technical, and translational challenges must be overcome before these platforms can achieve widespread clinical application. Future research should focus on optimizing nanoparticle size, composition, surface characteristics, biodegradability, and biodistribution to maximize therapeutic efficacy while minimizing toxicity. Achieving these goals will require a more comprehensive understanding of how nanoparticle physicochemical properties influence interactions with immune cells, vascular tissues, and infected organs. Detailed in vitro and in vivo investigations are needed to characterize nanoparticle–host interactions throughout the different stages of sepsis and septic shock. In particular, studies should establish the comparative efficacy, pharmacokinetics, biodistribution, safety, and long-term toxicological profiles of emerging hollow nanoparticle platforms using clinically relevant preclinical models. Emphasis should be placed on determining whether these nanoparticles can restore immune competence during the immunosuppressive phase of sepsis, an important but largely understudied aspect of disease progression.

Continued advances in biomimetic, hybrid, and phospholipid-based hollow nanoparticle technologies may further enhance targeting specificity, biocompatibility, and therapeutic performance. Multifunctional nanoparticle systems capable of simultaneously scavenging RONS, neutralizing microbial toxins, modulating dysregulated immune responses, and delivering therapeutic agents may provide a more comprehensive approach to addressing the complex pathophysiology of septic shock. Ultimately, rigorous clinical investigations will be required to determine whether the promising therapeutic effects observed in experimental models translate into meaningful improvements in patient survival, organ function, and long-term clinical outcomes. Continued progress in nanoparticle engineering, translational research, and clinical evaluation is expected to accelerate the development of next-generation hollow nanoparticle therapeutics for the management of septic shock.

## 8. Conclusions

Septic shock is characterized by a dysregulated host response to infection that progresses from an initial hyperinflammatory phase to immune dysfunction, multi-organ failure, and, in many cases, death. Consequently, there is an urgent need for therapeutic strategies capable of restoring immune homeostasis while minimizing tissue injury. Hollow nanoparticles have emerged as promising nanotherapeutic platforms because of their ability to target multiple pathological processes involved in septic shock. Depending on their composition and design, these nanoparticles can scavenge excessive RONS, modulate dysregulated immune signaling pathways, neutralize bacterial toxins and pathogen-associated molecular patterns, and suppress excessive inflammatory responses. In addition, certain hollow nanoparticle systems possess intrinsic antimicrobial activity or enhance the delivery and efficacy of antimicrobial agents, further expanding their therapeutic potential.

Despite these advances, several challenges continue to limit the clinical translation of hollow nanoparticle-based therapies. As septic shock evolves from an early hyperinflammatory phase to a subsequent immunosuppressive state, a single therapeutic strategy may not be effective throughout the entire course of disease. Although many hollow nanoparticles have been developed to attenuate excessive inflammation, their ability to restore immune competence during the immunosuppressive phase of sepsis remains poorly understood. Therefore, future studies should evaluate the stage-specific effects of hollow nanoparticle therapies and investigate combination approaches capable of addressing the distinct phases of immune dysregulation that characterize septic shock.

Additional barriers to clinical implementation include uncertainties regarding biodistribution, clearance mechanisms, immunogenicity, hemocompatibility, long-term safety, large-scale manufacturing, batch-to-batch reproducibility, regulatory approval, and cost-effectiveness. Overcoming these challenges will require the development of safer and more precisely engineered nanoparticle systems, standardized manufacturing and characterization protocols, and comprehensive preclinical and clinical evaluation. Additionally, combination strategies incorporating complementary nanoparticle platforms may provide opportunities to simultaneously target multiple pathogenic mechanisms involved in septic shock.

In conclusion, hollow nanoparticles represent a promising and rapidly evolving class of nanomedicines with the potential to address several unmet therapeutic needs in septic shock. Although significant scientific, regulatory, and translational challenges remain, continued interdisciplinary research integrating nanotechnology, immunology, materials science, and critical care medicine is expected to accelerate their development. Successful resolution of these challenges may ultimately enable hollow nanoparticle-based therapies to emerge as effective adjunctive or standalone treatments for septic shock, a condition for which targeted therapeutic options remain limited.

## Data Availability

The original contributions presented in this study are included in the article. Further inquiries can be directed to the corresponding author.

## Abbreviations

The following abbreviations are used in this manuscript:

RONS	Reactive oxygen and nitrogen species
IL-1	Interleukin-1
MODS	Multiorgan dysfunction syndrome
SIRS	Systemic inflammatory response syndrome
PRRs	Pattern recognition receptors
PAMPs	Pathogen-associated molecular patterns
DAMPs	Damage-associated molecular patterns
IFN	Interferon
IL-6	Interleukin 6
IL-12	Interleukin-12
STAT3	Signal transducer and activator of transcription 3
TNF	Tumor necrosis factor
MDR	Multi-drug resistant
ROS	Reactive oxygen species
ATP	Adenosine triphosphate
eLNPs	lipid-containing empty lipid nanoparticles
OX40L	OX40 ligand
CD40	Cluster of Differentiation 40
LPS	Lipopolysaccharide
TLR	Toll-like receptor
MYD88	Myeloid Differentiation Primary Response 88
NF-κB	Nuclear factor kappa-light-chain-enhancer of activated B cells
JNK	c-Jun N-terminal kinase
TRAM	Translocating Chain-Associating Membrane Protein
TRIF	TIR-domain-containing adapter-inducing interferon
TGF-b	Transforming growth factor beta
PEG	Polyethylene glycol
NO	Nitric oxide
H-MnO_2_	hollow manganese dioxide
mPDA	mesoporous polydopamine
GlyNPs	polymeric glycine nanoparticles
IL-10	Interleukin-10
CD8	Cluster of differentiation 8
PD-1	Programmed Cell Death Protein 1
CD3	Cluster of differentiation 3
CD28	Cluster of differentiation 28
BM-AuNPs	Biomimetic gold nanoparticles coated with bacterial outer membrane vesicles
ARDS	Acute respiratory distress syndrome
MS	Manganese silicate
NLRP3	NOD-like receptor protein 3
RBC	Red blood cell
MRSA	Methicillin-resistant Staphylococcus aureus
FDA	Food and Drug Administration
SOFA	Sequential Organ Failure Assessment
MAP	Mean Arterial Pressure
MAPK	mitogen-activated protein kinase
